# The Emergence and Evolution of Porcine Sapelovirus: Insights from Genomic Surveillance and Recombination Analysis in China

**DOI:** 10.3390/v18091012

**Published:** 2026-09-14

**Authors:** Jie Tao, Benqiang Li, Jinghua Cheng, Ying Shi, Lilei Lv, Qi Li, Huili Liu

**Affiliations:** 1 Institute of Animal Sciences and Veterinary Medicine, Shanghai Academy of Agricultural Sciences, Shanghai 201106, China; livia_taojie@126.com (J.T.); huteng2010@saas.sh.cn (B.L.); zero5cheng@saas.sh.cn (J.C.); shiying@saas.sh.cn (Y.S.); lvlilei@saas.sh.cn (L.L.); liqi@saas.sh.cn (Q.L.); 2 Shanghai Key Laboratory of Agricultural Genetic Breeding, Shanghai 201106, China; 3 Shanghai Engineering Research Center of Pig Breeding, Shanghai 201302, China

**Keywords:** PSV, genomic surveillance, recombination, evolution, co-infection

## Abstract

Porcine sapelovirus (PSV) is an emerging enteric pathogen associated with diarrheal disease in pigs, yet its epidemiological characteristics and genomic diversity in China remain inadequately characterized. In this study, we systematically compiled epidemic data on all Chinese PSV strains reported in domestic and international literature and constructed a geographic distribution map of PSV across China. Analysis of co-infection patterns revealed that PSV frequently co-occurs with PEDV, PAstV, and PKoV. A total of 74 PSV Chinese strains were retrieved from GenBank for genomic characterization. No distinct regional clustering was observed among the prevalent strains; however, a remarkably high recombination rate of 85.14% was identified, with recombination breakpoints predominantly concentrated near the 2A gene region. Temporal phylogenetic analysis indicated that avian sapeloviruses and PSV shared a similar evolutionary timeline (1985), with PSV-1 emerging earlier than PSV-2. Within the PSV-1 subtype, the German strain exhibited the earliest divergence (1997), followed by Chinese strains (1985), then Korean and American strains (2005). These findings underscore the importance of sustained genomic surveillance and highlight the need for further investigation into the pathogenicity of circulating recombinant strains and their roles in polymicrobial infections, which is critical for developing effective control strategies.

## 1. Introduction

The family *Picornaviridae* comprises a diverse group of small, non-enveloped, positive-sense single-stranded RNA viruses, many of which are significant pathogens in humans and animals [1]. The classification of picornaviruses has undergone substantial revisions over time as molecular biology techniques have advanced. Before the establishment of the genus *Sapelovirus*, its members were often misclassified because of limited genomic data. For instance, viruses now recognized as sapeloviruses were initially grouped with enteroviruses or teschoviruses, mainly based on their clinical manifestations and the host species. This historical background is important for understanding the taxonomic development of the porcine sapelovirus (PSV). PSV was initially classified as group II porcine enterovirus (PEV). It was later distinguished through virus neutralization assays, which divided PEVs into 15 serotypes across three groups (I, II, and III) according to cytopathic effects (CPE) in porcine kidney cells, physicochemical characteristics, and host range [2,3]. PSV was first designated as PEV serotype 8 (PEV-8) within CPE type II and was subsequently assigned to the genus *Enterovirus* as PEV-A [3,4]. Distinct genomic features, including a type IV internal ribosomal entry site (IRES), leader protein (L protein), and conserved 2A protein, reveal that PEV-8 differs substantially from other enteroviruses [5]. Consequently, it was reclassified into the newly established genus *Sapelovirus* within the family *Picornaviridae* [6]. This genus currently comprises three members originating from simian, avian, and porcine hosts [7]. Avian and porcine sapeloviruses each comprise a single serotype, whereas simian sapeloviruses comprise three serotypes [8,9,10].

PSV infections in pigs can lead to a wide range of clinical manifestations, including encephalomyelitis, diarrhea, pneumonia, respiratory distress, and reproductive failure, although asymptomatic infections are frequently observed [11,12,13]. In sows, infection may result in fetal death, stillbirth, mummification, and congenital malformations [14]. PSV primarily colonizes porcine intestinal epithelial cells, and infected pigs may continue to shed the virus even after clinical recovery. Therefore, these animals can act as long-term reservoirs, contributing to the sustained circulation of PSV within herds. Co-infections are common, particularly with the porcine epidemic diarrhea virus (PEDV), porcine teschovirus (PTV), and porcine deltacoronavirus (PDCoV). Such co-infections may enhance disease severity through synergistic interactions [15,16,17]. Although PSV is frequently subclinical [18], its potential effects should not be overlooked.

Therefore, this study aimed to explore the epidemiology, genetic evolution, recombination dynamics, and selection pressures of PSV in China. It also systematically integrated nationwide surveillance data to highlight the continued increase in PSV prevalence and changing epidemiological patterns. In addition, our analysis identified the 2A gene region as a recombination hotspot and demonstrated that complex recombination events play a dominant role in shaping PSV evolution in China.

Taken together, these findings offer a comprehensive understanding of PSV dynamics and provide substantive guidance for the design of future surveillance strategies.

## 2. Materials and Methods

### 2.1. Data Collection for Epidemiological Analysis

Currently, few reports are available on the epidemiology of PSV, and no systematic studies have been performed. To systematically investigate the prevalence of PSV in China, we searched the PubMed and China National Knowledge Infrastructure (CNKI) databases for relevant literature on PSV epidemiology and viral isolation. From PubMed, we retrieved 12 papers [16,17,19,20,21,22,23,24,25,26,27,28], all of which were included in the analysis. From CNKI, 45 PSV-related papers were identified, among which 13 papers [29,30,31,32,33,34,35,36,37,38,39,40,41] were epidemiological surveys and thus met our inclusion criteria. For regions with multiple reports, the prevalence was recorded as the average. In addition, 1933 fecal samples from piglets with diarrhea between 2018 and 2025 were collected for the surveillance of swine diarrheal pathogens in Shanghai, China. These data were also integrated into the overall statistical analysis of PSV prevalence. Based on these data, we analyzed the temporal distribution of PSV prevalence in China, the proportional distribution of PSV among different age groups of pigs, and the temporal changes in the proportion of PSV-positive cases between healthy and diarrheic pig populations.

### 2.2. Recombination and Phylogenetic Tree Analysis

All available full-length PSV sequences from China (*n* = 74) were retrieved from GenBank and analyzed for recombination using the RDP software package (4.0) [42]. Recombination events were screened using seven detection algorithms (RDP, GENECONV, BootScan, MaxChi, Chimaera, SiScan, and 3Seq). For each algorithm, an average *p*-value (Av. *p*-Val) < 10^−3^ was considered evidence of recombination. Events supported by at least four algorithms with Av. *p*-Val < 10^−3^ were considered reliable recombination events. The homology and phylogenetic tree of the 74 PSV strains were analyzed based on the P1 capsid-coding region.

### 2.3. Divergence Time Tree

To evaluate the genetic relationships and evolution rate of PSV, the full-length sequences of the available PSV1, PSV2, avian sapelovirus, marmot sapelovirus, and PEVs were analyzed by a time tree using the maximum likelihood method in MEGA X (11.0) with 1000 bootstrap replicates [43]. Divergence times for all nodes were estimated using the RelTime with Dated Tips (RTDT) method [42]. The dataset included 54 viral strains, including nine PEVs, two marmot sapeloviruses, one avian sapelovirus, and 42 PSVs, collected between 1958 and 2025 (Supplementary Table S1). The PEV-1 strain (AB038528, red) was used as an outgroup.

## 3. Results

### 3.1. Current Epidemiological Status of PSV in China

**Figure 1:**
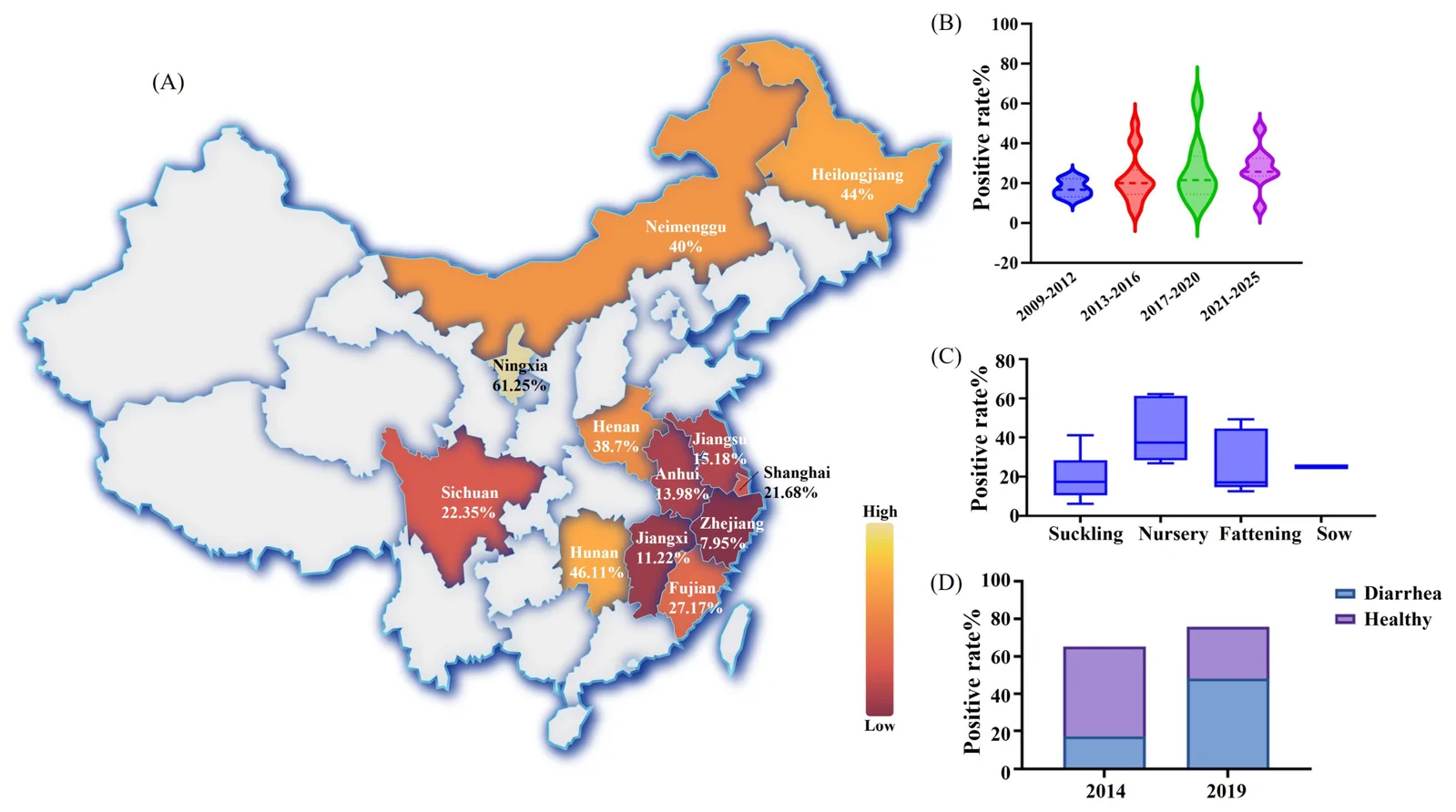
Epidemiological survey of Porcine Sapelovirus (PSV) in China. (**A**) Geographic distribution of PSV infection rates across Chinese provinces based on the available literature. (**B**) Trends in PSV prevalence across different sampling periods. (**C**) PSV infection rates stratified by pig age. (**D**) Comparison of PSV prevalence between herds with healthy pigs and herds with diarrhea.

Based on the statistical analysis of PSV prevalence data reported in the domestic and international literature, we constructed a prevalence map of PSV in China. The results indicated that the highest positivity rate was reported in Ningxia (61.25%), followed by Hunan (42.21–50.00%), Heilongjiang (44.00%), and Inner Mongolia (40.00%) (Figure 1A). Considerable variation was observed in other regions, with detection rates ranging from 22.83% to 55.96% in Henan, 12.50% to 29.90% in Sichuan, 5.55% to 22.30% in Anhui, 10.47% to 34.10% in Shanghai, and 8.75% to 20.00% in Jiangsu. Limited data from Zhejiang and Jiangxi revealed detection rates of 7.95% and 11.22%.

Over time, the prevalence of PSV in Chinese pig herds has shown a gradual increase, rising from 17.4% during 2009–2012 to 22.59% during 2013–2016, 25.66% during 2017–2020, and 27.69% during 2021–2025 (Figure 1B). When stratified by production stage, weaned piglets had the highest infection rate (43.35%), followed by fattening pigs (27.04%) and sows (25.00%), whereas suckling piglets showed the lowest rate (20.14%) (Figure 1C). PSV has also been frequently detected in healthy pigs. In 2014, the prevalence in healthy pigs (48.10%) was nearly three times higher than that in diarrheic pigs (17.20%), corresponding to a ratio of approximately 2.8:1. By 2019, this ratio had reversed to 1:1.7, suggesting a change in the epidemiological pattern (Figure 1D).

### 3.2. Coinfection of PSV and Other Diarrhea Pathogens

**Figure 2:**
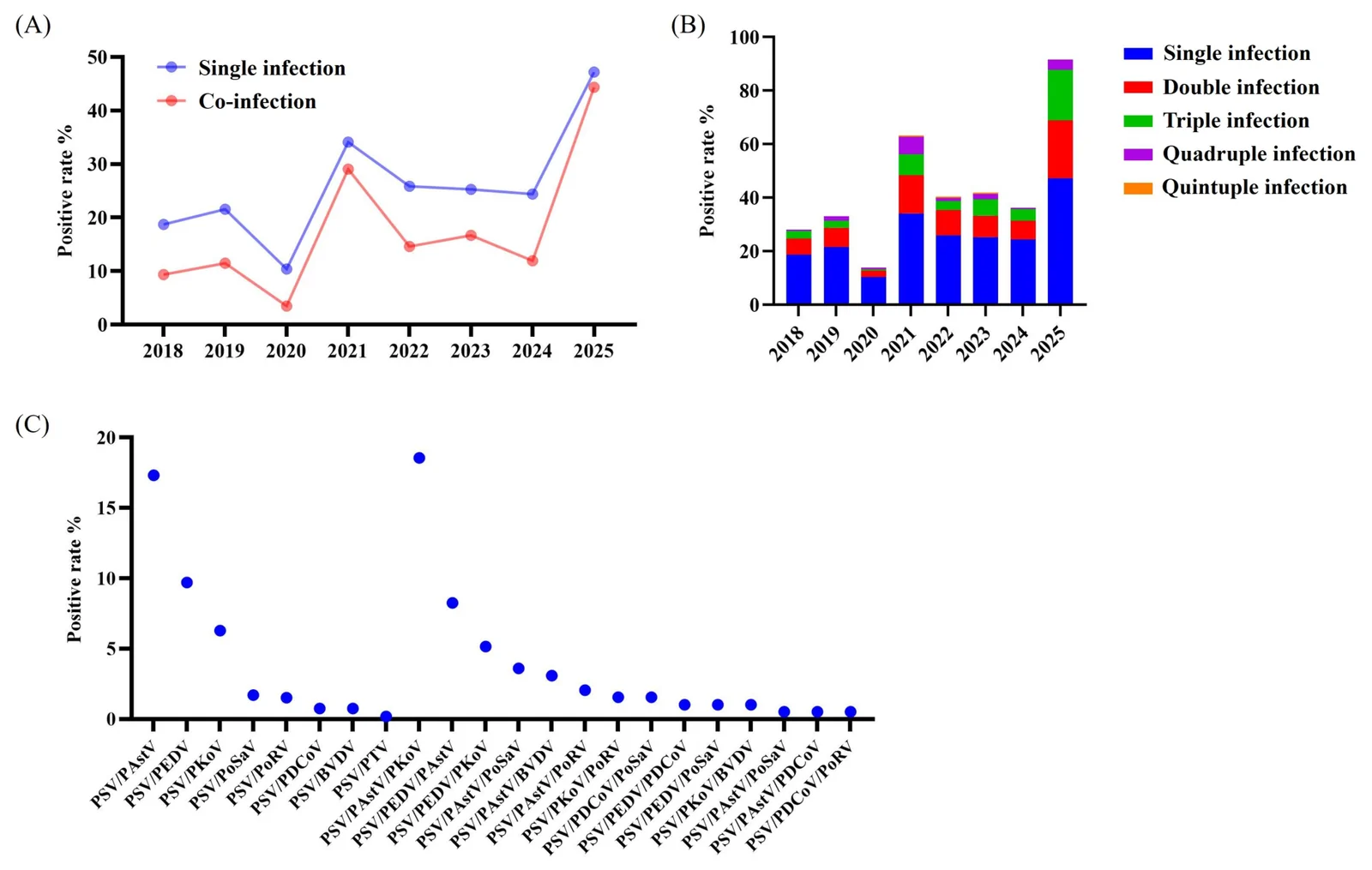
Profile of PSV co-infections in China. (**A**) Comparison of trends in single infections and co-infections. (**B**) Annual analysis of the PSV infection patterns. (**C**) Analysis of the PSV co-infection combinations.

In this study, based on 1933 diarrheal samples collected in our laboratory, we statistically analyzed the co-infection rates of PSV with other major diarrheal pathogens. The results showed that the trends of single and mixed PSV infections generally overlapped, with the annual single infection rate consistently remaining approximately twice that of mixed infections. From 2018 to 2025, both single and mixed PSV infection rates increased steadily (Figure 2A). By 2025, the two rates were nearly comparable, with single infections reaching 47.17% and mixed infections reaching 44.34%. Regarding infection patterns, the distribution was as follows: single infection (25.93%) > double infection (9.51%) > triple infection (5.83%) > quadruple infection (2.09%) > quintuple infection (0.18%) (Figure 2B). Notably, since 2023, the prevalence of triple infections has increased markedly and has nearly reached the level of double infections, reflecting the growing complexity of the PSV co-infection profiles.

The most frequent double infections were PSV/PAstV (17.33%), PSV/PEDV (9.71%), and PSV/PKoV (6.29%). The predominant triple-infection combinations were PSV/PAstV/PKoV (18.56%), PSV/PEDV/PAstV (8.25%), and PSV/PEDV/PKoV (5.15%) (Figure 2C). These findings suggest that PSV frequently co-occurs with PEDV, PAstV, and PKoV. Previous studies have shown that co-infection with PKoV and PEDV can worsen clinical symptoms in piglets, indicating potential synergistic interactions between these viruses [44]. Whether PSV/PEDV co-infection produces a similar synergistic effect remains unclear and requires further investigation. Clarifying this potential interaction is important for understanding the clinical significance of PSV in co-infected herds.

### 3.3. Genetic Analysis of Prevalent PSV Strains in China

**Figure 3:**
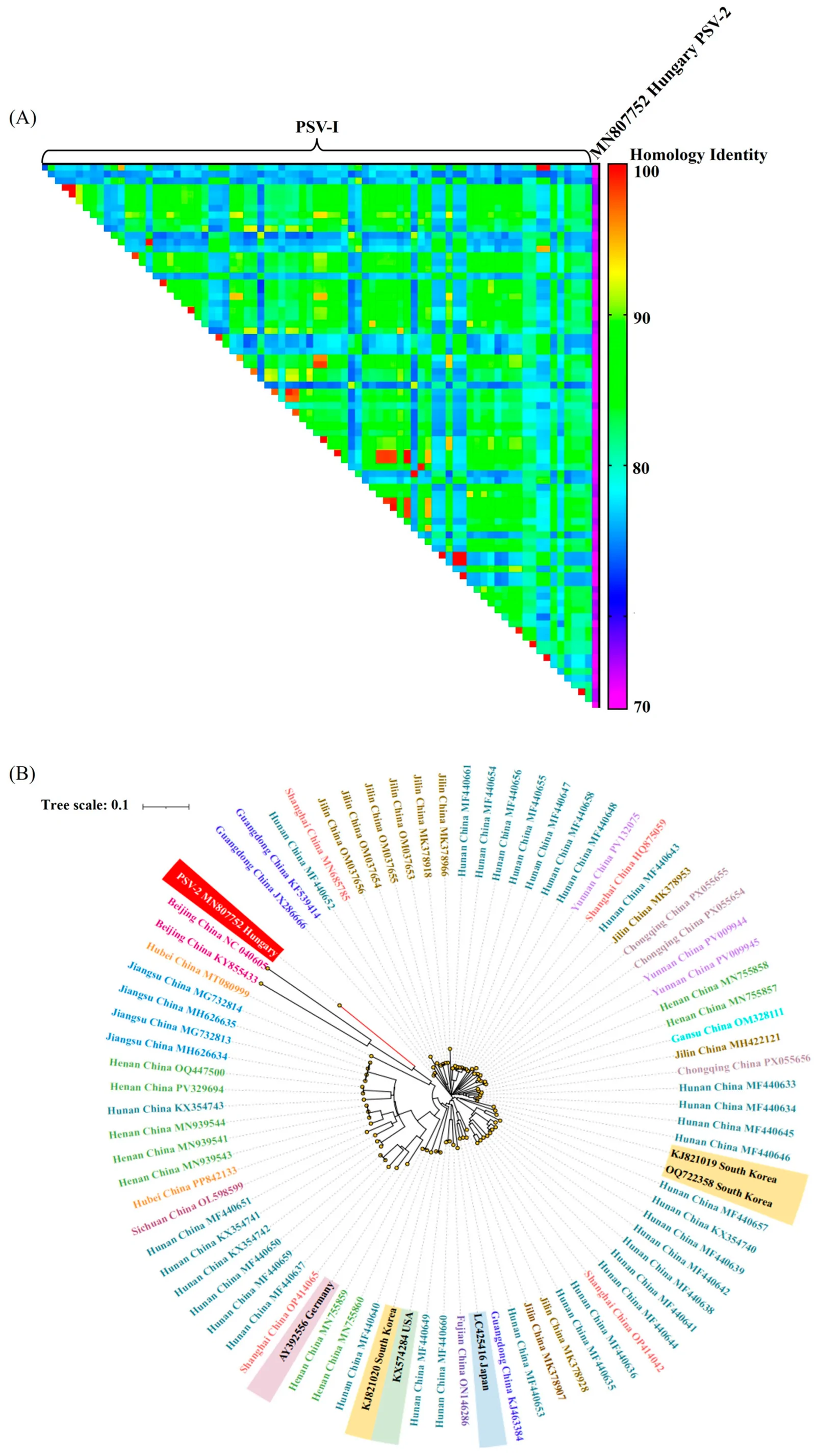
Genetic relatedness analysis of epidemic PSV strains in China. (**A**) Nucleotide identity heatmap. Seventy-four Chinese PSV sequences and seven foreign strains were obtained from GenBank (https://www.ncbi.nlm.nih.gov/) (accessed on 9 September 2025). Sequence alignment and pairwise identity analyses were performed using the DNASTER Lasergene (7.1). The percentage identity is shown using the color scale on the right. (**B**) Phylogenetic analysis. It was reconstructed using full-length nucleotide sequences with the Neighbor-Joining method in MEGA X. The Kimura 2-parameter model and 1000 bootstrap replicates were used. Strains from the same region are marked with the same font color, and foreign strains are indicated by different background colors.

We retrieved 74 available Chinese PSV sequences from GenBank and performed genetic and evolutionary analyses based on the P1 capsid-coding region. Homology analysis divided the sequences into four similarity ranges: <70.00% (2.47%), 70.00–80.00% (38.37%), 80.00–90.00% (54.14%), and >90.00% (5.01%) (Figure 3A). All Chinese PSV-1 strains shared more than 70% whole-genome identity, whereas their similarity to PSV-2 reference strains ranged from 63.84% to 67.18%, showing a clear genetic separation between the two genotypes (Figure 3A).

Phylogenetic analysis showed that the 74 Chinese PSV strains, together with isolates from South Korea, Japan, and the United States, clustered within PSV-1 and were clearly separated from marmot sapelovirus and PSV-2 strains. Chinese strains did not form clear geographic clusters. For example, the Hunan strains appeared across different branches and were mixed with isolates from other regions. Similarly, the four Shanghai strains were scattered across the PSV-1 clade rather than forming a single cluster. Interestingly, one Hubei strain (MT080999), which has been classified as PSV-1 in some previous studies based on partial gene analysis [17], showed a closer affinity to PSV-2 in our phylogenetic tree (Figure 3B). To further examine this strain, we calculated the pairwise sequence identities. The complete genome of MT080999 shared 71.50% identity with the PSV-2 reference strain (e.g., KJ627638) and 73.20% identity with the PSV-1 reference strain (e.g., AY392556). In the P1 region, which determines the serotype, MT080999 shared 78.10% identity with PSV-2 and 75.40% with PSV-1. Further, the recombination analysis indicated a high-confidence recombination event involving MT080999, PV009944 (major parent), and OQ722358 (minor parent), with breakpoints located at 1134–3450 nt (Supplementary Figure S1). The PV009944 strain was isolated from Yunnan in China in 2023, and OQ722358 was isolated from South Korea in 2022, both belonging to the PSV-1 genotype.

When compared with global references, several Chinese strains showed close relationships with international isolates. Three South Korean strains clustered with the Hunan isolates. One Japanese strain (LC425416) was grouped with a Fujian strain (ON146286). The German strain (AY392556) was closest to the Shanghai strain (OP414065), and one US strain (KX574284) clustered with a Hunan strain (MF440649) (Figure 3B). These results suggest that Chinese PSV strains are genetically linked to international isolates, which may reflect cross-border spread or shared evolutionary origins, highlighting the complex global circulation of PSV.

### 3.4. Genetic Recombination in Prevalent PSV Strains in China

**Figure 4:**
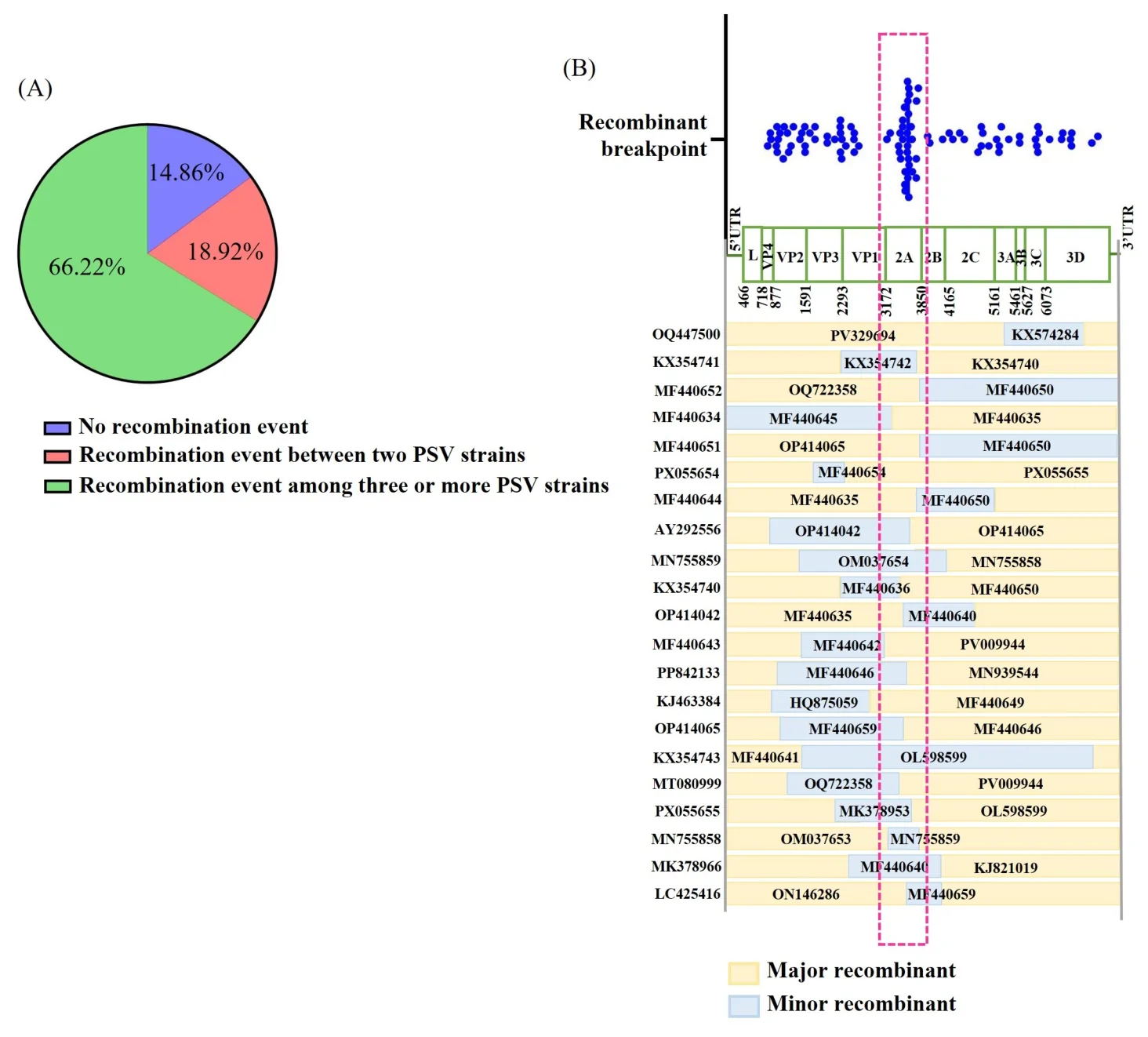
Prediction and analysis of recombination trends in the PSV. Full-length genome sequences from 74 Chinese PSV isolates were analyzed for recombination using the RDP 4.0 software. The proportions of recombination events are shown in (**A**,**B**). Significant recombination breakpoints identified from these events are summarized and shown in a scatter plot (top panel, (**B**)). Twenty-one high-confidence recombination events, consistently detected by seven algorithms (RDP, GENECONV, BootScan, MaxChi, Chimaera, SiScan, and 3Seq), were selected for detailed analysis. Their recombination maps are shown in the bottom panel, aligned with the PSV genome schematic and the breakpoint scatter plot for comparison. The dashed frame indicates a region with dense clustering of recombination breakpoints.

Among the 74 Chinese PSV strains, viral recombination analysis showed that 85.14% of the strains had recombination events, while 14.86% showed no evidence of recombination (Figure 4A). Among recombinant strains, 18.92% involved recombination between two parental strains, and 66.22% involved three or more parental sequences (Figure 4A), indicating that complex recombination is common in PSV strains in China. In Hunan Province, only two of the 29 isolates showed no recombination. Four involved two parental strains, and 23 involved three or more strains, further supporting this pattern.

Breakpoint analysis showed clear clustering in the 2A gene region (Figure 4B), suggesting that this region may act as a hotspot for PSV recombination and evolution. To further describe the recombination patterns, 21 high-confidence recombination events were selected for analysis (Supplementary Table S2). All seven algorithms showed an Av. *p*-Val < 10^−3^ for these strains, indicating strong support for the recombination. The results showed that 61.90% of events occurred in the genomic region from the L–VP1 segment to the 5′ portion of the 2A gene, with a clear breakpoint cluster in the 2A region. This supports the idea that the 2A gene is a recombination hotspot (Figure 4). The recombination breakpoint positions and Av.

### 3.5. The Divergence Genetic Evolution of PSV

**Figure 5:**
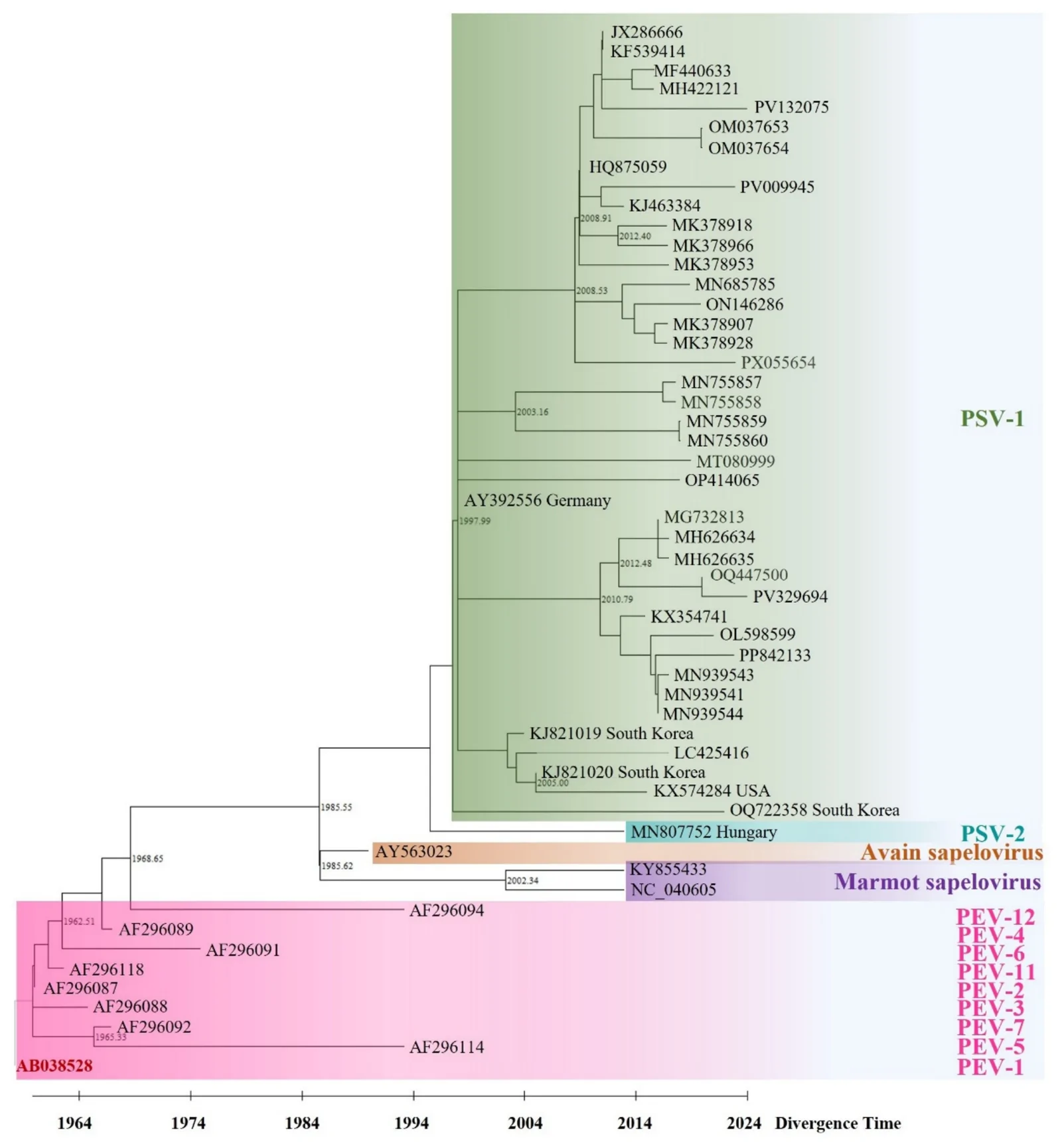
Temporal origin and evolutionary analysis of PSV. A time-scaled phylogenetic tree was reconstructed using MEGA X software with the RelTime with Dated Tips method based on the full-length genome sequences of 54 viruses, including 42 porcine sapeloviruses, nine porcine enteroviruses (PEV), two marmot sapeloviruses, and one avian sapelovirus. The sampling year for each sequence was used as a calibration constraint. The estimated divergence time for each major branch is shown.

A divergence time tree was constructed to investigate the origin and evolutionary history of PSV. The results indicated that PEV-1 emerged first around 1958, followed by other genotypes of PEV in the following order: PEV-2 (1960) > PEV-11 (1963) > PEV-3 (1965) > PEV-7 and PEV-4 (1967) > PEV-6 (1976) > PEV-5 and PEV-12 (1994) (Figure 5). Within the genus Sapelovirus, the sapelovirus lineage, formerly PEV-8, appeared around 1968, whereas avian and marmot sapeloviruses emerged in 1985 and 2002, respectively. PSV diverged in 1985, with PSV-1 emerging before PSV-2. Among the PSV-1 strains, the earliest sequenced isolate included in our dataset was a German strain (AY392556) collected in 1997. This was followed by a Chinese isolate (KF631220), referenced in the phylogenetic analysis of Lan et al. and sampled in 2003. It is important to distinguish between the year when PSV was first isolated and characterized in China (2009) and the collection date of the earliest Chinese sample, which was later sequenced and deposited in GenBank (2003). The latter provides molecular evidence that PSV was likely circulating in China prior to its formal discovery in 2009, which is consistent with the high genetic diversity observed in the initial study. Other PSV-1 strains were isolated from Japan, South Korea, and the United States in 2005. In contrast, PSV-2 was first reported in Hungary in 2013 [44], and no further detections have been reported since, suggesting that PSV-1 remains the predominant subtype worldwide.

## 4. Discussion

This study provides a comprehensive update on the epidemiology, genetic evolution, and recombination dynamics of porcine sapelovirus (PSV) in China by integrating nationwide surveillance data with newly generated local monitoring results and in-depth genomic analyses. Our findings highlight the increasing prevalence of PSV, the shifting patterns of its clinical associations, and the critical role of complex recombination, particularly within the 2A gene region, in shaping its evolutionary trajectory.

The steady increase in PSV prevalence in Chinese pig herds over the past decade and a half, from 17.4% in 2009–2012 to 27.69% in 2021–2025, is a salient finding. This upward trend is consistent with reports from other countries [44,45,46] and likely reflects a combination of true viral spread, improved surveillance, and heightened awareness. More importantly, our data reveal a notable epidemiological shift: the ratio of PSV detection in healthy pigs to that in diarrheic pigs changed from approximately 2.8:1 in 2014 to 1:1.7 in 2019. Although this observation should be interpreted cautiously because of potential heterogeneity among the compiled studies, it raises the compelling hypothesis that the pathogenic potential of PSV or its synergistic interactions with other pathogens may be evolving. The high infection rate among weaned piglets (43.35%) suggests that this group is particularly vulnerable, possibly because of waning maternal antibodies and the physiological stress associated with weaning, making them a key target for future intervention strategies.

Our analysis of co-infection patterns, based on 1933 diarrheic samples, reveals an increasingly complex landscape. The dramatic increase in mixed infections, from negligible levels to nearly 44.34% by 2025, and the emergence of triple infections at rates approaching those of double infections, highlight the growing challenge of polymicrobial diseases in swine herds. PSV was most frequently detected in co-infection with PAstV, PEDV, and PKoV. This is of particular concern because previous research has demonstrated that co-infection with PKoV can exacerbate PEDV pathogenicity [47]. Although a similar synergistic effect between PSV and PEDV remains to be experimentally demonstrated, our epidemiological data strongly suggest that PSV is not merely a passive bystander but an active participant in the complex etiology of porcine diarrhea. This finding emphasizes the need for multi-pathogen diagnostic panels and a holistic approach to disease management.

The most significant contribution of this study is the systematic characterization of PSV recombination. Our analysis of 74 full-length Chinese PSV genomes demonstrates that recombination is not an occasional event but a pervasive evolutionary force, with 85.14% of strains showing evidence of recombination. The predominance of complex, multi-parent recombination events (66.22%) is striking and indicates a highly dynamic viral population. Crucially, we identified the 2A gene region as a major recombination hotspot. This finding has considerable biological significance in light of the known functions of the 2A protein in picornaviruses. Beyond its structural role, the 2A protein, often functioning as a protease (2Apro), serves as a key “security protein” that counteracts host antiviral responses and creates a favorable environment for viral replication [48,49]. Enterovirus 71 (EV71) 2Apro cleaves key innate immune signaling molecules such as MAVS and components of the NLRP3 inflammasome, thereby facilitating immune evasion and directly modulating viral replication and virulence [50]. Notably, recent evidence has demonstrated that the PSV 2A protein itself is a critical virulence factor that induces mitochondria-dependent apoptosis in host cells, and this pro-apoptotic activity is strictly dependent on its protease active site [51]. Given these functional attributes, we propose that the 2A gene region is not merely a passive recombination hotspot but may represent an active site of adaptive evolution. Through frequent recombination, PSV may rapidly generate diverse 2A protein variants with different capacities to suppress host immunity or modulate pathogenicity, thereby gaining a selective advantage under host immune pressure. This hypothesis, linking recombination hotspots to functional selection, provides a compelling framework for understanding the sustained evolution and changing clinical impact of PSV. The identification of this hotspot also provides a clear focus for future mechanistic studies, such as the use of reverse genetics systems to investigate how specific 2A sequences or structures influence recombination frequency and viral fitness.

Furthermore, our phylogenetic analysis illustrates the extensive genetic diversity of PSV-1 in China and its close relationships with international strains. This finding suggests a complex history of global viral circulation, potentially driven by the international trade of pigs or pig products. The case of the Hubei strain MT080999 is particularly instructive. Although its P1 region shares higher identity with PSV-2, its genome is a mosaic resulting from recombination between PSV-1 parental strains. This example clearly demonstrates how recombination can confound genotype assignment based on single gene fragments or even whole-genome identity, highlighting the necessity of recombination-aware phylogenetic approaches for accurate viral classification.

This study has several limitations. First, the epidemiological analyses are based on a compilation of published data, which may be affected by sampling biases and heterogeneous methodologies across different studies and time periods. Second, our identification of the 2A gene as a recombination hotspot is based on computational prediction and lacks experimental validation. Third, the divergence time tree is retrospective and dependent on the available collection dates, which may not perfectly reflect the true evolutionary timelines. Despite these limitations, our findings provide a robust framework for understanding PSV evolution and epidemiology in China.

In conclusion, this study demonstrates that PSV is a highly prevalent and continuously evolving pathogen in Chinese pig herds. Its increasing prevalence, changing clinical associations, and complex recombination dynamics, particularly in the 2A gene, underscore its potential threat to swine health. We advocate for sustained genomic surveillance, the development of vaccines that account for circulating recombinant strains, and further experimental research into the pathogenicity of these emerging variants and their synergistic roles in co-infections. Such integrated efforts are essential for developing effective control strategies to mitigate the impact of PSV on the global swine industry.

## Data Availability

The original contributions presented in this study are included in the article and Supplementary Material. Further inquiries can be directed to the corresponding authors.

## Supplementary Materials

The following supporting information can be downloaded at: https://www.mdpi.com/article/10.3390/v18091012/s1, Figure S1: Recombination analysis of MT080999 strain; Table S1: The dataset included 54 viral strains, including nine PEVs, two marmot sapeloviruses, one avian sapelovirus, and 42 PSVs, collected between 1958 and 2025; Table S2: Recombination breakpoint positions and average *p* values (Av. *p* Val) from seven detection algorithms for 21 high-confidence recombination events.
