# Supplementary material for: The Emergence and Evolution of Porcine Sapelovirus: Insights from Genomic Surveillance and Recombination Analysis in China
Source: Viruses. 2026 Sep 14;18(9):1012. doi: 10.3390/v18091012 (PMC13612067; doi:10.3390/v18091012)
Supplement: Supplementary file 1 [file viruses-18-01012-s001.zip › viruses-4555668-Suplementary Figure S1.pdf]

Supplementary Figure S1. Recombination analysis of MT080999 strain.

（A）The phylogenetic tree was contructed based on the full-length sequences using the neighbor-joining method in MEGA X. (B) Viral recombination was analyzed and potential recombinant breakpoins (1134-3450nt) were identified by RDP4.0.BOOTSCAN on the basis of pairwise diatance with 1000 bootstrap replicates. (C) Recombination events in the MT080999 strain were scanned using seven recombination detection algorithms (RDP, GENECONV, BootScan, MaxChi, Chimaera, SiScan, and 3Seq) integrated in RDP4. For each algorithm, an average P-value (Av. P-Val) < 10^{-3} was considered as supporting recombination. When at least four algorithms yielded Av. P-Val < 10^{-3}, the event was deemed a reliable recombination event.

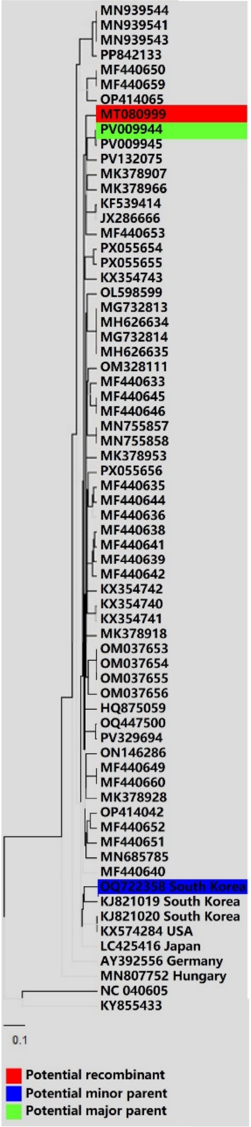

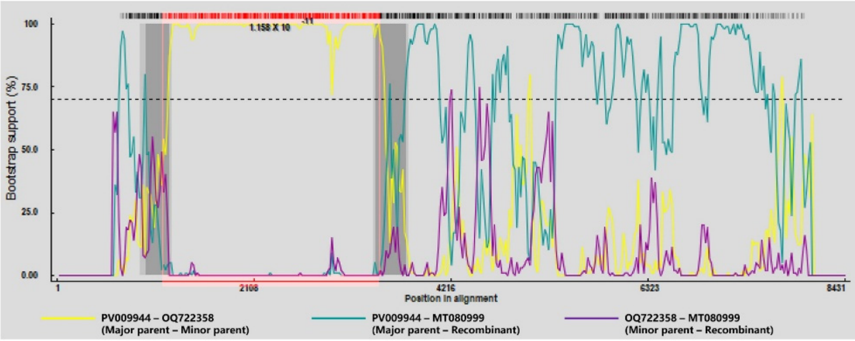

Confirmation Table

Methods	# seqs detected in	Av. P-Val
RDP	1	1.351×10^{-16}
GENECONV	1	6.591×10^{-04}
BootScan	1	6.130×10^{-11}
MaxChi	1	1.194×10^{-14}
Chimaera	1	2.020×10^{-17}
SiScan	1	1.360×10^{-25}
3Seq	1	8.121×10^{-12}
